# Dry powder inhalers and the right things to remember: a concept review

**DOI:** 10.1186/s40248-015-0012-5

**Published:** 2015-04-03

**Authors:** Roberto W Dal Negro

**Affiliations:** National Center for Respiratory Pharmacoeconomics and Pharmacoepidemiology - CESFAR, Verona, Italy; Research & Clinical Governance, Verona, Italy

**Keywords:** Dose consistency, Dry powder inhalers, Inhalation devices, Inspiratory flow resistance, Respiratory drug effectiveness

## Abstract

Dry powder inhalers (DPIs) are widely and increasingly used in clinical practice because they represent a substantial advancement in inhalation technology. The effectiveness of a powdered drug to inhale depends on the inspiratory flow rate generated by the patient and on the turbulence produced by the intrinsic resistance of the DPI. While the inspiratory flow is variable with the patient’s ability and conditions, the turbulence is differently sized within each device because depending of its technical design. There are higher - medium-, and low-resistance devices. With low-resistance DPIs, the disaggregation and the microdispersion of the drug highly depend on the patient’s inhalation airflow rate, because the role of the resistance-induced turbulence is obviously negligible in these cases. This flow-rate dependency is minimized in the presence of a sufficient regimen of turbulence as in the case of medium-resistance DPIs. Both the disaggregation and the micro-dispersion of the powdered drug are optimized in these circumstances even in the absence of a maximal inspiratory flow rate.

The low resistance DPIs should not be regarded as the best performer DPIs because their intrinsic low-resistance regimen requires a higher inspiratory airflow rate and effort, which frequently cannot be achieved by subjects suffering from a disease-induced airflow limitation.

Only when the ratio between the inhalation flow rate and the DPI intrinsic resistance is balanced, the speed of the particulate**,** the distribution of the drug within the lung, and the variability of the effective inhaled dose are optimized.

## Introduction

The delivery of pharmacological agents by inhalation is a critical issue in obstructive airway diseases (Bronchial Asthma and Chronic Obstructive Pulmonary Disease (COPD)). It represents a major component of their therapeutic management because inhaled drugs are targeting the lungs directly, consenting a lower dose together with a quick onset of action, and a better therapeutic index [1,2].

Several effective molecules have been developed in the last decades, but their true effectiveness in real life can be affected and modulated substantially by the device used for inhalation [3-5].

## Review

### The ideal device

An increasing number of inhalation devices have been engineered in the same period, either for single or combined molecules. However, it was assumed since long ago that the ideal device should be:

1. *effective*: such as, able to consent the inhalation of a sufficient fraction of drug with a particle size ≤ 6 μ, independently of the patient’s inspiratory flow;
2. *reproducible*: such as, able to always consent the inhalation of the same drug amount, also in terms of its respirable fraction;
3. *precise*: such as, able to consent to know at any moment the amount (or the n. of doses) of the drug remaining in the device, and whether or not the inhalation was correctly performed: thus the need for providing DPIs of a “*dose counter”* and of a “*double-dosing protection counter”,* in order to avoid a further inhalation if the patient is unaware or not sure of having taken the previous one [6];
4. *stable*: such as, able to protect the drug(s) contained from the effects of temperature and/or humidity changes;
5. *confortable*: such as, easy to use in different circumstances (particularly in critical conditions), and possibly containing several doses of the drug(s) for a long-term use;
6. *versatile*: such as, it should consent the use of other drugs by inhalation;
7. *environmental compatible*, such as not containing chemical contaminants;
8. *affordable*: such as, of acceptable cost, and possibly rechargeable [2].

### The DPIs’ family

Independently of wet nebulizers, pocket devices can be basically grouped in three major classes: a) the Metered Dose Inhalers (MDIs), still largely used for single and combined molecules, and which need a propellant for the dose delivery; b) the Dry Powder Inhalers (DPIs), which do not require any propellant, and are increasingly prescribed for single and combined molecules; c) the Soft Mist Inhalers (SMIs), at present consisting in only one device for only one molecule (Respimat for Tiotropium bromide).

DPIs, even if widely variable in design, definitively represent a substantial improvement in the inhalation therapy because they fit the majority of the above mentioned requirements. In particular, they eliminate the use of propellants; simplify the inhalation technique; reduce the patient’s cooperation and improve the patient’s compliance to treatment; favour a higher deposition of drugs within the lungs; reduce the variability of the inhaled dose; reduce the incidence of both local and systemic side effects, and finally ameliorate the consistency of the dose and then the outcomes substantially [7-10]. Furthermore, most advanced DPIs also fitted the most sophisticated patients’ requirements in terms of minimization of the number of actions needed for preparing the actuation.

### Main characteristics of DPIs

Basically, DPIs can be differentiated according to their intrinsic resistive regimen, such as a constant which depends on the original constructive design of each device, and which is evaluated by measuring the extent of pressure drop across the device itself (Table 1).

**Table 1:** **The main classes of DPIs, based on their intrinsic resistance and pressure drop across the device**

	**Pressure drop across the device**
**Low resistance DPIs**	**<5 Mbar** 1/2 L/min −1
**Medium resistance DPIs**	**5-10 Mbar** 1/2 L/min −1
**High resistance DPIs**	**>10 Mbar** 1/2 L/min −1

When rewievig the most common DPIs used in clinical practice, the HandyHaler; the Easyhaler and the Twisthaler belong to the class of the higher-resistance devices, while the Turbohaler; the Accuhaler/Diskus, the Ellipta, the Novolizer, and the Genuair belong to the group of medium-resistance devices, and the Aerolizer and the Breezhaler belong to the class of low-resistance devices [11,12].

In general terms, the performance of each DPI can be affected by only two main driving forces: 1) the inspiratory flow generated by the patient, and 2) the turbulence produced inside the device, which uniquely depends by its original technical characteristics [1,11]. These are the only two factors able to affect the disaggregation of the powdered drug dose, the diameter of the particles to inhale, the consistency and the variability of the dose, substantially.

In particular, the inspiratory airflow generated by the patient represents the only active force (a passive force for the device) able to produce the micro-dispersion (even if differently sized for each device) of the powdered drug to inhale. On the other hand, the extent of the patient’s inspiratory airflow depends on the patient’s airway and lung conditions, and, partially, on the intrinsic resistive regimen of the device.

During an inspiratory manoeuvre, the right balance between these two forces represents the critical factor which decides the true effectiveness of the couple “molecule-device”. Higher the airflow, higher the powder dispersion generating a fine particulate, even if such a high airflow leads to a higher impaction losses in the proximal airways and, consequently, to a lower dose reaching peripheral airways [2,11]. On the other hand, a lower airflow consents a deeper lung deposition of the powdered drug, even if a too low airflow (as that one existing in the severest patients) can limit deposition by affecting powder disaggregation and dispersion.

Obviously, changes in these two forces can be achieved only by changing the airflow characteristics or the original DPI design.

In particular, when using a medium-resistance DPI, both the disaggregation and the micro-dispersion of the powdered drug are relatively independent of the patient’s inspiratory airflow because the driving force depending on the intrinsic resistance of the DPI itself is able to produce *per sè* the turbulence required for an effective drug microdispersion. In these cases, the speed of the particulate is lower, the distribution of the drug is much better within the lung, and the variability of the effective inhaled dose is quite lower, thus leading to a drug delivery which is more fitting to the corresponding original claim [13].

On the contrary, when using a low-resistance DPI, the only driving force for the disaggregation and the microdispersion of the drug to inhale is represented by the patient’s inhalation airflow rate (the role of the resistance-induced turbulence is obviously negligible in these cases), which only depends, even if at a different extent, on the patient’s airflow limitation and in addition, on his disease severity. As a consequence, the required regimen of turbulence can be achieved only by increasing the inhalation airflow, which nevertheless frequently represents the main critical limitation for airway obstructive patients. In these circumstances, the variability in the dose consistency is higher and the effective inhaled dose can be far from the original claim, also due to the higher oro-pharingeal impact of the powdered drug.

Actually, we are in the presence of a “conceptual misunderstanding” which is crucial for interpreting the events and for deciding which DPI is more convenient for the patient in real-life. In other words, the “low resistance DPIs” should not be mandatory associated to the concept of “the most effective DPIs” because just in these cases patients are required for a higher inspiratory performance, which frequently cannot be achieved by patients affected by a disease-induced airflow limitation.

Unfortunately, these concepts were not sufficiently clarified and popularized in general practice, and they were progressively neglected and practically left to the wrong simplistic interpretation of the term “low-resistance”, which spontaneously recalls *per sè* the principle of “easiness of use”, particularly in non expert prescribers. Also the reluctance of producers to better explain or characterize their device played a crucial role in neglecting these aspects.

Presumably, the real-life scenario is getting increasingly confusing in the near future because, further to the numerous DPIs already available for therapeutic purposes, some novel devices are entering the market. An increasing range of intrinsic resistance regimens should then be considered, because the DPIs’ performances will further vary between each other in terms of therapeutic effectiveness also substantially.

A technical review on DPIs currently available on the market has been recently carried out in order to compare in standard conditions (at a defined pressure point of 4 kPa) their intrinsic characteristics in terms of inspiratory device resistance, of inspiratory flow rate and corresponding pressure drop, and of their performance variability [11]. In total agreement with the concept previously mentioned, the low-resistance DPIs confirmed those requiring the highest inspiratory flow rates for consenting an effective actuation and those characterized by the highest variability in delivery of respirable fraction of the drug (Table 2).

**Table 2:** **Differences in intrinsic resistance and in inspiratory flow rate through the device of some of most commonly used DPIs**

	**Inspiratory DPI resistance (kPa** ^**0.5**^ **L/min)**	**Inspiratory flow rate (L/min)**
**Breezhaler®**	0.017	111
**Aerolizer®**	0-019	102
**Ellipta®**	0.027	74
**Novolizer®**	0.027	72
**Accuhaler/Diskus®**	0.027	72
**Genuair®**	0.031	64
**Nexthaler®**	0.036	54
**Turbohaler®**	0.039	54
**Handihaler®**	0.058	37

From [10].

In particular, the Breezhaler, which is the DPI device at present characterized by the lowest intrinsic resistance (such as, 0.017 kPa^0.5^ L/min), proved to require the highest inspiratory flow rate at an average of 111 L/min (min 102 and max 117 L/min). Moreover, it showed a mean pressure drop of 2.5-4 kPa, and a large variability in the dose delivery, by a standard deviation higher than 4% [11]. An equivalent performance has been assessed for other DPIs with the same intrinsic characteristics (i.e. the Aerolizer) (Table 2).

Other DPIs which are characterized by a medium intrinsic resistance consent a better performance from this point of view. Actually, the Novolizer; the Accuhaler/Diskus, the Genuair, which have an intrinsic resistance of 0.027; 0.027, and 0.031, respectively, confirmed to require a much lower inspiratory flow rate for an effective actuation (such as: 72; 72, and 64 L/min) (Table 2). The corresponding pressure drop was ranging 6.6-9.5 kPa, with the lowest rate of variability, by a standard deviation lower than 1% in the case of Genuair [11].

Finally, high resistance DPIs (ranging 0.035-0.058 kPa^0.5^ L/min) even if allowing a lower inspiratory flow rate, proved to affect particle generation and dispersion of powdered drug substantially [14,15].

## Conclusions

The critical factors driving the therapeutic effectiveness of a respiratory drug assumed through a DPI are represented by the constructive constants of the device and by the generation of an inhalation airflow rate sufficient to trigger the dose and disaggregate the drug, thus producing a particulate of optimal size, and able to reach the therapeutic targets within the airways.

It should be taken into account because a proportion of patients is unable to afford an effective inhalation in real life. Particularly in COPD, it mainly depends on limitations in their cognition performance [16], on their physical limitations, but also on their severe and persistent airway impairment. On the other hand, the extent of this phenomenon has been confirmed to change according to the prescribed DPI [16,17].

The particle size is another critical point for the effectiveness of treatment because it affects the respirable fraction and the lung deposition of the inhaled drug. The inhalation speed can also play a role because while small particles (1–2 μ in diameter) show a comparable effect independently of the inhalation speed, larger particles (3–6 μ in diameter) are more effective when inhaled at a lower speed [18-20]. This aspect has been overcome by the use of DPIs which are provided with a trigger valve which consents the release of the powdered drug only after a preset airflow rate (35 L/min) has been achieved [21,22,23].

In conclusion, the effectiveness of a powdered drug to inhale mainly depends on the inspiratory flow rate generated by the patient and on the intrinsic resistance of the DPI device. These two forces affect the dose utilization, both the powder disaggregation and dispersion, its penetration into the airways, and finally the efficacy of treatment.

An increasing number of DPIs are available for different drugs, and they are differently characterized in terms of their intrinsic constants, even if wrong assumptions are still diffuse concerning the therapeutical performance of DPIs in real life. In other words, while low-resistance DPIs are still regarded as the easiest and the most comfortable devices for the patient, they instead require a high inhalation airflow rate to the patient, not always achievable. The reason is that the role of the other possible force involved in drug deagglomeration (such as the DPI intrinsic resistance) is negligible in these circumstances, and the whole effect is merely depending on the patient’s high flow rate. Actually, when using this kind of DPIs, the patient has sometimes to repeat the inhalation manoeuvre in order to inhale completely the dose of the powdered drug in the capsule, particularly the most compromised patients.

Differently, medium-resistance DPIs require a lower inhalation flow rate to the patient because the turbulence generated by the intrinsic resistance regimen operating inside these devices contribute substantially to the drug deagglomeration and to the effective production of particulate, thus reducing the impaction losses in the upper airways and reducing the aerodynamic variability of the particulate itself.

Medium-resistance DPIs combine the advantage of requiring more achieveable flow rates with that of producing an effective respirable fraction of the drug to inhale. These characteristics are particularly relevant in terms of real-life utilization by compromised and flow-limited patients who can find in the generation of high inspiratory flow rates the major limit in the effectiveness of their respiratory treatment.
